# Assessing and harnessing updated polyketide synthase modules through combinatorial engineering

**DOI:** 10.21203/rs.3.rs-3157617/v1

**Published:** 2023-07-28

**Authors:** Katherine A. Ray, Joshua D. Lutgens, Ramesh Bista, Jie Zhang, Ronak R. Desai, Melissa Hirsch, Takeshi Miyazawa, Antonio Cordova, Adrian T. Keatinge-Clay

**Affiliations:** 1 Department of Molecular Biosciences, The University of Texas at Austin, Austin, TX; 2 Department of Chemistry, The University of Texas at Austin, Austin, TX

## Abstract

The modular nature of polyketide assembly lines and the significance of their products make them prime targets for combinatorial engineering. While short synthases constructed using the recently updated module boundary have been shown to outperform those using the traditional boundary, larger synthases constructed using the updated boundary have not been investigated. Here we describe our design and implementation of a BioBricks-like platform to rapidly construct 5 triketide, 25 tetraketide, and 125 pentaketide synthases from the updated modules of the Pikromycin synthase. Every combinatorial possibility of modules 2–6 inserted between the first and last modules of the native synthase was constructed and assayed. Anticipated products were observed from 60% of the triketide synthases, 32% of the tetraketide synthases, and 6.4% of the pentaketide synthases. Ketosynthase gatekeeping and module-skipping were determined to be the principal impediments to obtaining functional synthases. The platform was also used to create functional hybrid synthases through the incorporation of modules from the Erythromycin, Spinosyn, and Rapamycin assembly lines. The relaxed gatekeeping observed from a ketosynthase in the Rapamycin synthase is especially encouraging in the quest to produce designer polyketides.

## INTRODUCTION

**Figure 1.**
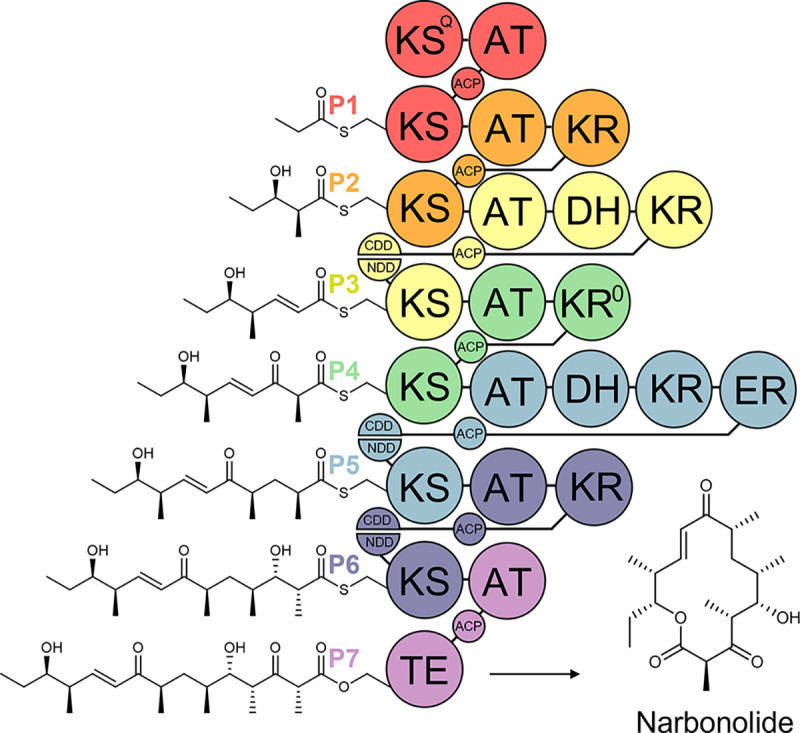
The Pikromycin PKS. The 7 modules (**P1-P7,** updated definition) of this polyketide assembly line collaborate to biosynthesize the heptaketide precursor of Pikromycin, Narbonolide. AT, acyltransferase; KR, ketoreductase; DH, dehydratase; ER, enoylreductase; ACP, acyl carrier protein; KS, ketosynthase; TE, thioesterase; KS^Q^, priming KS; KR^0^, epimerase, CDD & NDD, C- and N-terminal docking domain motifs.

Modular polyketide synthases (PKS’s) are multi-domain enzymatic assembly lines that biosynthesize many important medicines, such as the antibacterial Erythromycin and the antifungal Amphotericin (Figure 1)^1,2^. Each PKS consists of sets of domains known as modules that work together to extend and modify the growing polyketide. Minimally, a module contains an acyltransferase (AT) domain that selects an α-carboxyacyl extender unit, an acyl carrier protein (ACP) domain that receives and shuttles an extender unit, and a ketosynthase (KS) domain that catalyzes carbon-carbon bond formation between an ACP-bound extender unit and a KS-bound polyketide. A module may also harbor processing enzymes, such as a ketoreductase (KR) that reduces the KS-generated β-keto group, a dehydratase (DH) that eliminates the KR-generated β-hydroxyl group, and an enoylreductase (ER) that reduces the DH-generated *trans*-α,β-double bond^3^. This machinery can theoretically provide access to a wide swath of chemical space with great significance to material, synthetic, and medicinal chemistry.

For three decades, most attempts to engineer PKS’s have used the traditional module boundary immediately upstream of KS and have been unsuccessful^4^. However, a study of related aminopolyol synthases provided evidence that KS’s evolutionarily co-migrate with processing domains upstream of this boundary^5^. Engineering with the updated module boundary downstream of KS has been much more successful^6–9^. Whereas traditionally-engineered triketide lactone synthases rarely yield titers greater than 10 mg L^−1 10–12^, triketide lactone synthases engineered in our lab using the updated module boundary yield up to 791 mg L^−1 8,13^. The higher activity of the updated module is attributed to keeping together processing enzymes that introduce functionality at the α- and β-positions with KS’s that gatekeep for the introduced functionality^14^.

Although our lab has been successful implementing the updated module boundary in the construction of trimodular synthases that generate small triketide products^7,8^, the modularity of updated modules and thus the synthetic potential of polyketide assembly lines constructed from them remain essentially unknown. In constructing larger synthases that more thoroughly test the updated boundary, we considered many potential impediments: KS’s could gatekeep beyond the β-position^14^; protein-protein interactions between neighboring modules could be suboptimal^3^; introduced docking domains could associate more weakly than in their native context^15,16^; processing enzymes could ignore unnatural substrates^17^; polypeptide stoichiometries could be unbalanced^13^; and polypeptides could be degraded^13^. Although each of these factors could hamper the activities of engineered synthases, we hypothesized that the predominant impediments would be identified if enough synthases were investigated. Thus, we first aimed to engineer all possible triketide, tetraketide, and pentaketide synthases using modules from the Pikromycin synthase^18^.

A BioBricks-like platform was designed such that DNA fragments encoding updated modules could be ligated in an idempotent manner between regions encoding the first and last modules of the Pikromycin synthase (**P1** and **P7**). Each synthase is engineered to possess one fewer polypeptide than its number of modules, and each of its polypeptides harbors docking motifs from the Spinosyn synthase appropriate for the self-assembly of the synthase. Five **P1-X-P7**, 25 **P1-X-Y-P7**, and 125 **P1-X-Y-Z-P7** expression plasmids were constructed and transformed into a metabolically engineered strain of *E. coli* that activates PKS polypeptides and supplies them with methylmalonyl extender units. Polyketide products were detected from media extracts by mass spectrometry, and several were isolated for further characterization by NMR and crystallography. Anticipated molecules were observed from 60% of the triketide synthases, 32% of the tetraketide synthases, and 6.4% of the pentaketide synthases. Our analysis reveals KS gatekeeping and module-skipping to be the major impediments to constructing functional synthases. The platform was also used to engineer hybrid synthases using updated modules from the Erythromycin, Spinosyn, and Rapamycin synthases. The substrate promiscuity displayed by a module from the Rapamycin assembly line is especially encouraging towards realizing the common goal of producing designer polyketides.

## RESULTS

### Combinatorial expression plasmids

**Figure 2.**
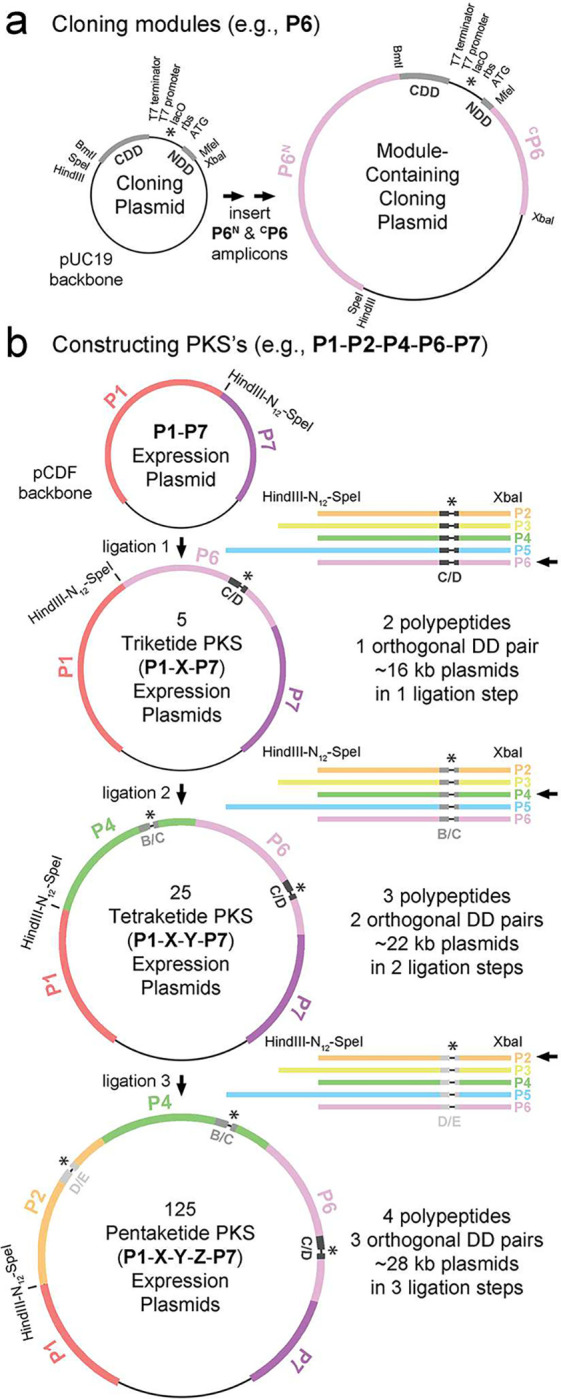
Platform for cloning modules and constructing PKS expression plasmids. a) Cloning Plasmids were made by inserting synthetic DNA between the HindIII and XbaI sites of pUC19. The insertion of amplicons encoding the N- & C-terminal portions of a module, such as the 6^th^ module of the Pikromycin synthase (**P6**), places cognate CDD and NDD docking motifs between its ACP and KS domains. b) The **P1-P7** Expression Plasmid was made by inserting DNA encoding **P1**, HindIII-N_12_-SpeI, and **P7** between the KpnI and AvrII sites of pCDF-1b. Module-encoding DNA cut from the Cloning Plasmids with HindIII and XbaI can be sequentially inserted to construct synthases like **P1-P2-P4-P6-P7** (Figure S1, Table S1).

A platform was designed so that module-encoding DNA, maintained on a cloning plasmid, could be idempotently inserted into an expression plasmid during synthase construction (Figure 2)^19^. Cloning plasmids were generated from pUC19 by inserting synthetic DNA encoding a T7 terminator, a T7 promoter, a *lac* operator, a ribosomal binding site, and C- and N-terminal docking domains from the Spinosyn PKS (the CDD/NDD’s of SpnB/SpnC, SpnC/SpnD, and SpnD/SpnE; ATG was used for the NDD start codons) (Figure S1)^15,16,20^. DNA encoding the N- and C-terminal portions of modules 2–6 of the Pikromycin synthase (**P2-P6**) was PCR-amplified from *Streptomyces venezuelae* ATCC 15439 and inserted between the SpeI and BmtI sites and MfeI and XbaI sites, respectively (Table S1). On the amino acid level, the XbaI site introduces an alanine and serine in the flexible region between ACP and CDD, and the MfeI site encodes a proline and isoleucine at the start of the KS domain where these residues are highly conserved. The expression plasmid was constructed by inserting DNA encoding **P1** and **P7** between the KpnI and AvrII sites of pCDF-1b. At the junction of these modules (10 residues downstream of the PikKS1 GTNAH motif and 11 residues downstream of the PikKS6 GTNAH motif), HindIII and SpeI sites were engineered to enable the insertion of HindIII/XbaI-digested, module-encoding DNA from cloning plasmids into the HindIII/SpeI-digested expression plasmid. On the amino acid level, this introduces residues at the updated module boundaries (KLAPAPTS at the junction downstream of **P1** and SS at the other junctions), which are known to be highly tolerant to insertions^21^. On the DNA level, the HindIII and SpeI sites are preserved upstream of the inserted module, and a nonfunctional XbaI/SpeI site is formed downstream of the inserted module.

The platform was first employed to construct 5 triketide synthases (**P1-X-P7**) by inserting DNA encoding **P2-P6**, each split by the SpnC/SpnD docking domains. Next, 25 tetraketide synthases (**P1-X-Y-P7**) were constructed by further inserting DNA encoding **P2-P6**, each split by the SpnB/SpnC docking domains. Finally, 125 pentaketide synthases (**P1-X-Y-Z-P7**) were constructed by further inserting **P2-P6**, each split by the SpnD/E docking domains.

### Polyketides from PKS’s constructed from the Pikromycin synthase modules

**Figure 3.**
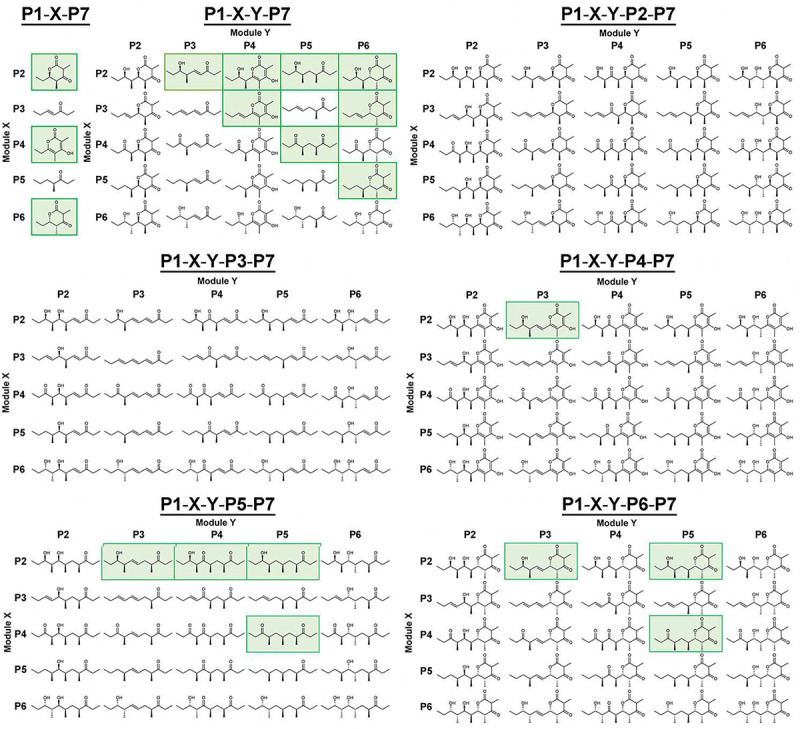
Polyketides anticipated and observed from the P1-X-P7, P1-X-Y-P7, and P1-X-Y-Z-P7 synthases. Green background indicates products observed by high-resolution mass spectrometry (Table S2).

**Figure 4.**
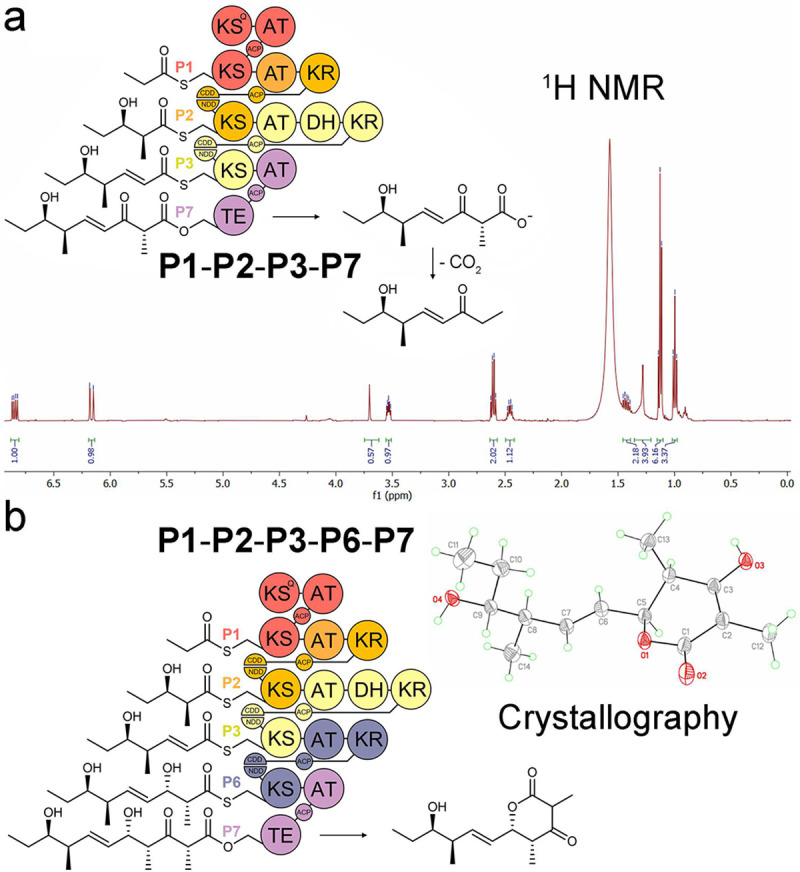
Polyketide products characterized by NMR and crystallography. a) ^1^H NMR spectrum of the **P1-P2-P3-P7** product. b) Crystal structure of the **P1-P2-P3-P6-P7** product.

*E. coli* K207–3 cells transformed with the triketide-pentaketide synthase expression plasmids were incubated in shake flasks at 19 °C^22^. After 7 days, ethyl acetate extracts of the cultures were analyzed by LC/MS. Anticipated products were detected from 3 triketide synthases (**P1-P2-P7**, **P1-P4-P7**, and **P1-P6-P7**), 8 tetraketide synthases (**P1-P2-P3-P7**, **P1-P2-P4-P7**, **P1-P2-P5-P7**, **P1-P2-P6-P7**, **P1-P3-P4-P7**, **P1-P3-P6-P7**, **P1-P4-P5-P7**, and **P1-P5-P6-P7**), and 8 pentaketide synthases (**P1-P2-P3-P4-P7**, **P1-P2-P3-P5-P7**, **P1-P2-P4-P5-P7**, **P1-P2-P5-P5-P7**, **P1-P4-P5-P5-P7**, **P1-P2-P3-P6-P7**, **P1-P2-P5-P6-P7**, and **P1-P4-P5-P6-P7**) (Figure 3). These products were validated by high-resolution mass spectrometry (Table S2 and Figure S2). Several were purified for further characterization by NMR (the products of **P1-P2-P3-P7**, **P1-P2-P4-P7**, **P1-P5-P6-P7**, **P1-P2-P3-P4-P7**, and **P1-P2-P3-P6-P7**) and crystallography (the product of **P1-P2-P3-P6-P7**) (Figures 4 and S3-S14).

### Module performance within engineered synthases

**P3-P6** were observed to function with non-native modules upstream of them, and **P2-P5** were observed to function with non-native modules downstream of them. However, modules often did not function as expected. Their behavior in different contexts was characterized to identify impediments to their modularity.

**P2** was observed to function in the 2^nd^ position (**P1-P2-P7**, **P1-P2-P3-P7**, **P1-P2-P4-P7**, **P1-P2-P5-P7**, **P1-P2-P6-P7**, **P1-P2-P3-P4-P7**, **P1-P2-P3-P5-P7**, **P1-P2-P4-P5-P7**, **P1-P2-P5-P5-P7**, **P1-P2-P3-P6-P7**, **P1-P2-P5-P6-P7**) but not in the 3^rd^ or 4^th^ positions. **P3** was observed to function in the 2^nd^ position (relatively small peaks were observed for the anticipated products of **P1-P3-P4-P7** and **P1-P3-P6-P7**), and in the 3^rd^ position when preceded by **P2** (**P1-P2-P3-P7**, **P1-P2-P3-P4-P7**, **P1-P2-P3-P5-P7**, **P1-P2-P3-P6-P7**), but not in the 4^th^ position. **P4** was observed to function in the 2^nd^ (**P1-P4-P7**, **P1-P4-P5-P7**, **P1-P4-P5-P5-P7**, **P1-P4-P5-P6-P7**), 3^rd^ (**P1-P2-P4-P7**, **P1-P3-P4-P7**, **P1-P2-P4-P5-P7**), and 4^th^ positions (**P1-P2-P3-P4-P7**). **P5** was observed to function in the 2^nd^ (**P1-P5-P6-P7**), 3^rd^ (**P1-P2-P5-P7**, **P1-P4-P5-P7**, **P1-P2-P5-P5-P7**, **P1-P4-P5-P5-P7**, **P1-P2-P5-P6-P7**, **P1-P4-P5-P6-P7**), and 4^th^ positions (**P1-P2-P3-P5-P7**, **P1-P2-P4-P5-P7**, **P1-P2-P5-P5-P7**, **P1-P4-P5-P5-P7**), and was the only module that was observed to function downstream of another copy of itself (**P1-P2-P5-P5-P7** and **P1-P4-P5-P5-P7**). **P6** was observed to function in the 2^nd^ (**P1-P6-P7**), 3^rd^ (**P1-P2-P6-P7**, **P1-P3-P6-P7**, **P1-P5-P6-P7**), and 4^th^ positions (**P1-P2-P3-P6-P7**, **P1-P2-P5-P6-P7**, **P1-P4-P5-P6-P7**). No synthase with **P6** immediately upstream of **P2-P6** was functional.

Most of the engineered synthases produce significant levels of shunt products (Table S3). For example, in addition to its anticipated pentaketide product, **P1-P2-P3-P6-P7** generates the tetraketide products made by **P1-P3-P6-P7**, **P1-P2-P3-P7**, and **P1-P2-P6-P7** as well as the triketide products made by **P1-P2-P7** and **P1-P6-P7**.

### Hybrid synthases

**Figure 5.**
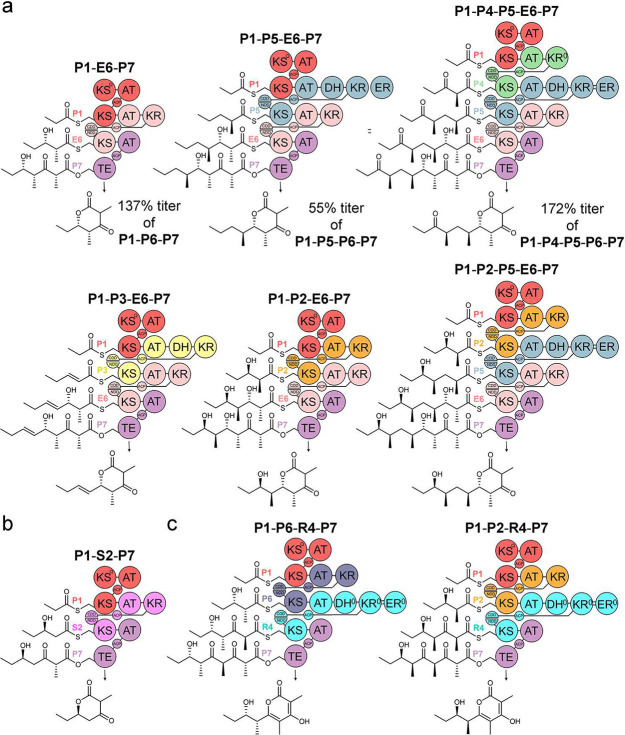
Hybrid synthases. a) The 6^th^ module of the Erythromycin synthase (**E6**), functionally equivalent to **P6,** behaves equivalently to **P6** in hybrid synthases. The productivities of the 3 hybrid synthases in top row are compared with their non-hybrid counterparts (Data S1). b) The 2^nd^ module of the Spinosyn synthase (**S2**) collaborates with Pikromycin modules in **P1-S2-P7** to yield the expected triketide lactone. c) As no Pikromycin module was observed to function immediately downstream of **P6**, the 4^th^ module of the Rapamycin synthase (**R4**), hypothesized to contain a more substrate-permissive KS, was placed downstream of **P6** in **P1-P6-R4-P7**. The anticipated tetraketide was produced. That **P1-P2-R4-P7** also produced its anticipated tetraketide reveals that RapKS4 can accept stereoisomeric intermediates with opposite stereochemistries at the γ- and δ-carbons.

We also sought to employ the platform to construct and assess the activities of hybrid synthases in which the updated modules from the Pikromycin synthase collaborate with those from other synthases (Figure 5). To determine whether modules that are functionally equivalent in natural synthases behave equivalently within engineered hybrid synthases, the 6^th^ module of the Erythromycin PKS (**E6**) was swapped for **P6** in functional **P6**-containing synthases to generate **P1-E6-P7**, **P1-P2-E6-P7**, **P1-P3-E6-P7**, **P1-P5-E6-P7**, **P1-P2-P5-E6-P7**, and **P1-P4-P5-E6-P7** (Figure 5a). Each of these hybrid synthases produces its expected polyketide. When the productivities of **P1-E6-P7**, **P1-P5-E6-P7**, and **P1-P4-P5-E6-P7** were compared with those of their non-hybrid counterparts, **P1-E6-P7** and **P1-P4-P5-E6-P7** were actually observed to generate higher polyketide titers (137% and 172%, Data S1). Similarly, the insertion of the 2^nd^ module of the Spinosyn PKS (**S2**) between **P1** and **P7** yielded the synthase **P1-S2-P7**, which synthesized its anticipated triketide lactone (Figure 5b).

In a recent KS gatekeeping study, most of the KS’s from the Rapamycin synthase were noted to possess an aromatic residue at Position 2 characteristic of KS’s that gatekeep less stringently^14^. Since this aromatic residue (phenylalanine in RapKS4 from the 4^th^ module of the Rapamycin synthase, **R4**) can nonspecifically interact with diverse polyketide intermediates, modules from the Rapamycin synthase may be particularly useful in accessing designer polyketides^23^. As no Pikromycin module was observed to accept an intermediate from **P6**, the ability of **R4** to do so within the tetraketide synthase **P1-P6-R4-P7** was tested (Figure 5c). Indeed, this synthase yields its anticipated product. The triketide synthase generated during its construction, **P1-R4-P7**, is also functional. A further test of the substrate tolerance of **R4** was made through the construction of **P1-P2-R4-P7**. The intermediate presented to RapKS4 in this synthase is a stereoisomer of the intermediate presented to RapKS4 in **P1-P6-R4-P7**, containing oppositely-oriented γ-methyl and δ-hydroxy substituents. Indeed, **P1-P2-R4-P7** also produces its anticipated tetraketide.

### Modeling natural intermediates into KS substrate tunnels

**Figure 6.**
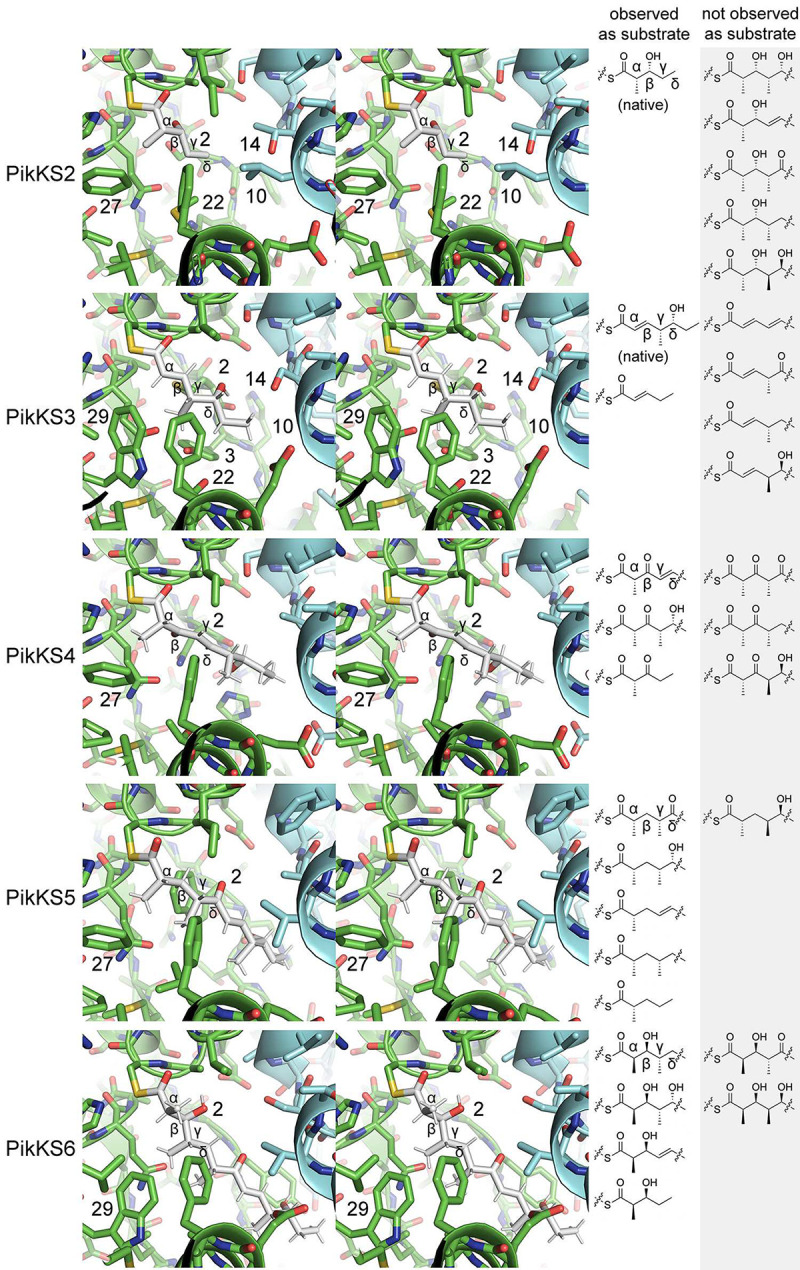
Intermediates accepted by the KS domains of the Pikromycin PKS. On the left, stereodiagrams show how natural polyketide substrates may be bound to the reactive cysteines of PikKS2-PikKS6 (structures generated by AlphaFold). The α-, β-, γ-, and δ-carbons of the intermediates as well as important gatekeeping residues are labeled (Hirsch et al., 2021). On the right, intermediates observed to be accepted or not observed to be accepted by KS’s within the engineered synthases are tabulated.

Modeling was performed to help understand the gatekeeping activities of KS’s employed in this study (PikKS2-PikKS6, SpnKS2, EryKS6, RapKS4) (Figures 6 and S18). First, AlphaFold predictions were obtained for each homodimeric KS^24^. Next, the coordinates and restraint files for polyketide substrates were generated using the program Sketcher^25^. Finally, they were positioned with the program Coot^26^ in conformations equivalent to those observed in acyl-KS structures (PDB Codes 2BUI, 2GFY, 2IX4, 6ROP, 7UK4; N-C_α_-C_β_-S and O-C-C_α_-C_β_ dihedral angles were kept within the experimentally observed ranges, except for the α/β-unsaturated PikKS3 intermediate, where O-C-C_α_-C_β_ = 0°)^14,27–31^.

## DISCUSSION

While our lab has employed the updated module boundary to engineer trimodular synthases that outcompete equivalent synthases engineered using the traditional module boundary^7,8,13^, and other labs have used it to modify larger synthases^9,32,33^, the utility of the updated module boundary has not been systematically examined. In this study, we sought to test the limits of PKS engineering with updated modules and identify impediments to programming synthases that biosynthesize designer polyketides. Our platform enabled the rapid construction of the expression plasmids for 5 triketide, 25 tetraketide, and 125 pentaketide synthases using the updated modules from the Pikromycin synthase and docking domains from the Spinosyn synthase.

Analysis of the culture extracts of *E. coli* K207–3 transformed with these plasmids revealed anticipated products for 60% of the triketide synthases, 32% of the tetraketide synthases, and 6.4% of the pentaketide synthases as well as shunt products for the majority of the synthases. The nature of the shunt products is revealing. For example, although **P1-P2-P3-P6-P7** generates its anticipated pentaketide, it also generates the tetraketides produced by **P1-P3-P6-P7**, **P1-P2-P3-P7**, and **P1-P2-P6-P7**. This apparent module-skipping naturally occurs in the Quartromicin synthase, in which the final domain of the assembly line is an ACP that is hypothesized to collaborate with the upstream KS to generate a hexaketide as well as the KS of the previous module to generate a pentaketide, both of which are components of Quartromicin^34,35^. In many of the synthases engineered here, the noncovalent associations of CDD/NDD pairs from the Spinosyn synthase may inadequately prevent ACP’s from accessing more than one upstream KS.

The most significant impediment to engineering is KS gatekeeping beyond the β-carbon^14,36,37^. Of the 5 tetraketide synthases that contain PikKS2 in the 3^rd^ position and the 25 pentaketide synthases that contain PikKS2 in the 4^th^ position, none yielded their anticipated product. Of the 5 tetraketide synthases that contain PikKS3 in the 3^rd^ position and the 25 pentaketide synthases that contain PikKS3 in the 4^th^ position, only **P1-P2-P3-P7** yielded its anticipated product. In the aforementioned KS gatekeeping study, we investigated how residues at 32 positions in the KS substrate tunnel help select for chemistries within polyketide intermediates^14^. Diketide-accepting KS’s like PikKS2 possess relatively large residues at Positions 2, 10, 14, and 22 that make favorable interactions with the tail of a diketide but sterically exclude longer intermediates (Figure 6). PikKS3 possesses a serine at Position 14 that can form a hydrogen bond with the d-δ-hydroxy group of its natural triketide intermediate but cannot make an equivalent hydrogen bond with an intermediate lacking a d-δ-hydroxy group. The ethyl tail of the triketide may fit in a hydrophobic pocket adjacent to the glycine in Position 10 not accessible to the tails of longer intermediates.

PikKS4 and PikKS6 were observed to less stringently gatekeep for substituents beyond the β-carbon (Figure 6). PikKS4 naturally accepts an intermediate containing a *trans-*double bond between its γ- and δ-carbons. Additionally, it was observed to accept an intermediate without γ- and δ-substituents in **P1-P4-P7**, **P1-P4-P5-P7**, **P1-P4-P5-P5-P7**, and **P1-P4-P5-P6-P7** and intermediates containing an l-γ-methyl group and a d-δ-hydroxyl group in **P1-P2-P4-P7** and **P1-P2-P4-P5-P7**. PikKS6 naturally accepts an intermediate containing an l-γ-methyl group and δ-methylene. Additionally, it was observed to accept an intermediate without γ- and δ-substituents in **P1-P6-P7**, an intermediate containing an l-γ-methyl group and a d-δ-hydroxyl group in **P1-P2-P6-P7**, and intermediates containing a γ,δ-*trans*-double bond in **P1-P3-P6-P7** and **P1-P2-P3-P6-P7**.

PikKS5 may be the least stringent gatekeeper of the Pikromycin KS’s (Figure 6). It naturally accepts an intermediate containing an l-γ-methyl group and a δ-keto group. Additionally, it was observed to accept an intermediate without γ- and δ-substituents in **P1-P5-P6-P7**, intermediates containing an l-γ-methyl group and a d-δ-hydroxyl group in **P1-P2-P5-P7** and **P1-P2-P5-P6-P7**, an intermediate containing a γ,δ-*trans*-double bond in **P1-P2-P3-P5-P7**, and intermediates containing an l-γ-methyl group and a δ-methylene group in **P1-P2-P5-P5-P7** and **P1-P4-P5-P5-P7**. The only module from which it was not observed to accept an intermediate is **P6**. The tryptophan at Position 2 of PikKS5 may help it nonspecifically interact with diverse polyketide intermediates^14^. When a tryptophan was introduced at this position in EryKS3, its substrate scope was observed to increase dramatically^23^.

Nearly 6 decades ago, Celmer noted that carbon skeletons of Erythromycin, Pikromycin, and related macrolide antibiotics possess similar stereochemical patterns^38^. While the basis for this is still being elucidated, part of the answer resides in the specificities of KS substrate tunnels. The KS’s of synthases that produce macrolide antibiotics have apparently evolved not only to recognize the chemistries at the α- and β-positions set by enzymes in the same module but also chemistries beyond the β-position set by enzymes in previous modules. While complementary interactions between KS’s and features beyond the β-position would increase the productivity of an assembly line, from an engineering standpoint modules containing these more substrate-specific KS’s are less desirable than modules containing KS’s that gatekeep only at the α- and β-positions.

The first 12 modules of the Rapamycin synthase synthesize the “variable region” of Rapamycin, and their highly identical KS’s possess an aromatic residue (F or Y) at Position 2 that is characteristic of less stringent gatekeepers^14,39^. The next 2 modules help synthesize the “constant region” of Rapamycin, and their KS’s do not possess the aromatic residue. The equivalent constant regions of Rapamycin, FK506, and WDB002 tightly bind eukaryotic prolyl isomerases, while their variable regions target a second eukaryotic protein – mammalian Target Of Rapamycin, calcineurin, and centrosomal protein 250, respectively^39^. The modules that synthesize the variable regions may be more portable and facilitate the evolution of motifs that target new secondary proteins. Indeed, a method that accelerates recombination within the Rapamycin synthase genes gave rise to synthases with 1–6 fewer module(s) and 1 more module that produce Rapamycin derivatives containing anticipated changes to the variable region in good yields^40^.

After employing the described platform to generate functional hybrid synthases containing modules from the Erythromycin and Spinosyn synthases, we attempted to incorporate a module from the Rapamycin synthase. Since none of the modules from the Pikromycin synthase was observed to accept intermediates from **P6**, we positioned the 4^th^ module of the Rapamycin synthase, **R4**, immediately downstream of **P6** within **P1-P6-R4-P7**. In this functional tetraketide synthase, RapKS4 accepts an intermediate with a d-oriented γ-methyl substituent. It is the only KS in this study observed to do so. PikKS3, PikKS5, and PikKS6 naturally accept intermediates with l-oriented γ-methyl substituents and may have evolved to interact with those methyl groups in their l-orientations. RapKS4 is apparently quite tolerant to γ- and δ-substituents since it accepts an intermediate without γ- and δ-substituents in **P1-R4-P7** as well as stereoisomeric intermediates with oppositely oriented γ- and δ-substituents in **P1-P6-R4-P7** and **P1-P2-R4-P7**. If modules from the Rapamycin synthase are permissive to intermediates with diverse chemistries beyond the β-carbon and yield the chemistries anticipated of them, they could play prominent roles in engineering PKS’s to yield designer polyketides.

The first assembly lines constructed with the described platform only contain modules from the Pikromycin synthase since we were concerned engineering would be impeded by adverse protein-protein interactions between modules, especially those of different synthases. However, hybrid synthases were observed to be highly functional. The 6 synthases in which **P6** was substituted by **E6** (**P1-E6-P7**, **P1-P2-E6-P7**, **P1-P3-E6-P7**, **P1-P5-E6-P7**, **P1-P2-P5-E6-P7**, and **P1-P4-P5-E6-P7**) show similar activities, suggesting that, although **P6** and **E6** are structurally distinct, their differences do not prevent them from equivalently collaborating with an upstream **P1**, **P2**, **P3**, **P5**, or a downstream **P7**. One could argue that evolutionary relationships between modules of PKS’s that synthesize macrolide antibiotics confer greater success rates to hybrid synthases comprised of them. However, the observed collaboration between modules of the Pikromycin synthase with modules of the more evolutionarily-distant Spinosyn and Rapamycin synthases suggests that the structurally compatibility of modules is not a major concern for PKS engineers.

In the synthases constructed here, the ordered assembly of polypeptides was mediated by 3 Class 1a Spinosyn CDD/NDD pairs. They substituted for other Class 1a pairs (in **P3** and **P5**) and Class 1b pairs (in **P6** and **S2**) as well as enabled the splitting of modules naturally encoded on one polypeptide (in **P2**, **P4**, **E6**, and **R4**). While these docking motifs were observed to function quite well overall, they may not always function as anticipated. We hypothesize that, similar to the Quartromicin synthase^34^, module-skipping can occur when ACP’s are not restrained (e.g., through the association of CDD/NDD pairs downstream of them). Imbalanced polypeptide stoichiometries could cause this, since an excess of an upstream polypeptide would mean some CDD motifs are not paired with their cognate NDD motifs on the downstream polypeptide^13^. While peptide connections could be employed to covalently anchor ACP’s to downstream KS’s to prevent module-skipping, this would result in lower polyketide titers due to the poor expression of long polypeptides in *E. coli*^8,13^.

In this work a high-throughput, BioBricks-style platform was developed to combinatorially construct expression plasmids for engineered PKS’s. Through it, 29 triketide-pentaketide synthases, including hybrid synthases, were obtained that yielded their anticipated products at titers that enable their characterization by NMR and crystallography. As few natural short synthases appropriate for *in vivo* and *in vitro* studies have been discovered, the triketide-pentaketide synthases engineered here can serve as valuable model systems. Through analyzing the activities of these synthases, we identified KS gatekeeping as the most significant impediment to PKS engineering. We demonstrated that this may be circumvented by employing modules containing substrate-tolerant KS’s, such as those from the Rapamycin synthase. By combining updated modules from diverse synthases with platforms like the one described here, the goal of engineering PKS’s to synthesize designer commodity chemicals and medicines is being realized.

## Data Availability

Data supporting the findings of this work are available within the paper and its Supplementary Information files. A reporting summary for this article is available as a Supplementary Information file. Data generated and analyzed herein are available from the corresponding author upon request. The x-ray coordinates for the structure reported in this study were deposited at the Cambridge Crystallographic Data Centre (CCDC) under deposition number 2278377. These data can be obtained free of charge from CCDC via www.ccdc.cam.ac.uk/data_request/cif.
